# Numerical and experimental investigation of light trapping effect of nanostructured diatom frustules

**DOI:** 10.1038/srep11977

**Published:** 2015-07-09

**Authors:** Xiangfan Chen, Chen Wang, Evan Baker, Cheng Sun

**Affiliations:** 1 Mechanical Engineering Department, Northwestern University, Evanston, IL 60208, USA

## Abstract

Recent advances in nanophotonic light-trapping technologies offer promising solutions in developing high-efficiency thin-film solar cells. However, the cost-effective scalable manufacturing of those rationally designed nanophotonic structures remains a critical challenge. In contrast, diatoms, the most common type of phytoplankton found in nature, may offer a very attractive solution. Diatoms exhibit high solar energy harvesting efficiency due to their frustules (i.e., hard porous cell wall made of silica) possessing remarkable hierarchical micro-/nano-scaled features optimized for the photosynthetic process through millions of years of evolution. Here we report numerical and experimental studies to investigate the light-trapping characteristic of diatom frustule. Rigorous coupled wave analysis (RCWA) and finite-difference time-domain (FDTD) methods are employed to investigate the light-trapping characteristics of the diatom frustules. In simulation, placing the diatom frustules on the surface of the light-absorption materials is found to strongly enhance the optical absorption over the visible spectrum. The absorption spectra are also measured experimentally and the results are in good agreement with numerical simulations.

Solar radiation is considered as one of the most abundant supplies of free energy in nature. Thus, solar cells have been extensively studied and optimized to increase their efficiency and reduce the cost to transform solar energy into electricity. In particular, the emergence of the thin-film solar cells opens up promising potential in cost reduction^1^,^2^, but has limited energy conversion efficiency caused by the mismatch between the characteristic length scale associated with optical absorption and carrier transportation^3^,^4^,^5^. To address such needs, light-trapping technologies were developed to extend the effective path length for light traveling inside the thin film solar cell and increase the efficiency of photon collection^6^,^7^,^8^,^9^,^10^. A variety of light-trapping structures have been proposed and explored, examples include photonic crystal structures^11^, triangular or pyramid gratings^12^, nanoparticles^13^, nanowires^14^, nanocones^15^ and plasmonic nanostructures^16^. However, fabricating these nanophotonic light-trapping structures containing subwavelength patterns using traditional top-down nanofabrication processes can be rather expensive. Thus, cost-effective scalable manufacture of nanophotonic light-trapping structures remains a critical challenge.

Through millions of years of evolution, nature often presents its unique, but surprisingly elegant solutions that even surpass modern engineering designs^17^,^18^,^19^,^20^. For instance, photonic crystal structures are found to produce structural coloration in butterfly wings and antireflection coatings in moth eyes^19^. Among the creatures capable of photosynthesis in nature, phytoplankton accounts for approximately 50% of all photosynthetic activity on earth^21^,^22^,^23^. Diatoms are the most common type of phytoplankton that live in almost every aquatic environment on the earth^23^. They exhibit high solar energy harvesting efficiency partly due to their hard porous shell made of silica. The shell, also known as the frustule^24^, possesses a remarkably organized and hierarchical three dimensional porous exoskeleton, with pore diameters ranging from 50 nm to more than 1 μm^25^,^26^,^27^,^28^. The frustule has been optimized for the photosynthetic process through natural evolution^29^. The size of diatoms themselves ranges from a few microns to 1 mm depending on the species, which can be potentially applied for photonic devices at various scales^30^,^31^. In particular, utilizing the light trapping effect of the diatom frustules may offer a promising potential to achieve mass production of a naturally optimized scattering layer for thin photovoltaic devices.

Although light focusing and manipulation effects of diatom frustules have already been studied^32^,^33^,^34^,^35^,^36^, the physical principle underlying their unique light-trapping effect has remained unexplored. In this paper, we report a theoretical and experimental study on the photonic properties of the diatom frustules and explore the potential application in enhancing the light absorption in PTB7:PC_71_BM based low-bandgap active materials, which has been widely used in the thin-film solar cells^37^,^38^. Diatoms (*Coscinodiscus sp*.) are cultured and the silica frustules with a hierarchical micro-/nano-structure can be well preserved for experimental characterization. Numerical simulations suggest that the light scattering by the hierarchical frustule structure results in enhanced light absorption in the regions from 380 nm to 500 nm and from 650 nm to 800 nm. Experimental measurements are in a good agreement with the simulation results. These results reveal the light-trapping effect due to the presence of hierarchical micro-/nano-scaled features in the frustules and the potential to enhance light absorption in the thin film solar cells.

Each diatom species possesses its unique and often highly intricate frustule morphology. In previous studies, the valves of the diatom *Coscinodiscus sp*. have attracted much attention due to their radial symmetry with large and flat surfaces featuring well-organized multilevel pores, while other pennate diatoms are generally elongated with bilateral symmetry^23^. Based on the experimental study by Losic *et al*., the *Coscinodiscus sp*. frustule consists of a hierarchical structure with three constituting layers, which are named as cribellum, cribrum and the internal plate, respectively^25^,^26^,^27^,^28^. Each layer is a thin film consisting a hexagonal array of circular holes, as shown in Fig. 1. The simplified three-dimensional (3D) structure of the frustule is schematically illustrated in Fig. 1(b), in which the periodic boundary condition is chosen along the x and y direction to represent the hexagonal array of holes. The geometrical parameters are shown in Fig. 1(c). It is generally believed that the incoming sunlight undergoes strong scattering processes in the nanostructured frustule and subsequently gives rise to the light-trapping effect to enhance the photosynthesis efficiency of the natural diatoms.

To understand the underlying physical principle of the light-trapping effect of the diatom frustule, the rigorous coupled-wave analysis (RCWA) method is employed^39^. RCWA solves the Maxwell’s equations in Fourier space, which is better suited to study the scattering problem in periodic structures than the commonly used time domain solver^40^. Fig. 2(a) presents the general structure of a thin-film solar cell model, where the diatom frustules are placed on the top of a 50 nm PTB7:PC_71_BM layer, which acts as the light absorbing material. Experimental data for the refractive index of PTB7:PC_71_BM and silica (SiO_2_) are used in the simulations^37^,^41^, (Fig. S1, Supplementary Information), and the hole is filled with air (n = 1). The transmission and reflection spectra are calculated under plane wave incidence along the normal and oblique direction. The absorption spectrum is determined by *A(λ)* = 1 *–* *R(λ)* – *T(λ)*, where *A(λ)*, *R(λ)* and *T(λ)* denote for the normalized absorption, reflection, and transmission, respectively. Considering the unpolarized nature of the sunlight, the absorption spectrum is averaged among incident electric field polarized along x-axis and y-axis. The simulation results on the polarization-dependent characteristics can be found in the Supplementary Information.

Numerical simulations are performed to first evaluate the contribution to the light trapping effect from each constituting layer of the diatom frustule. RCWA simulated absorption spectra of the thin-film solar cell model with individual constituting layer, including cribellum, cribrum, and internal plate, are shown in Fig. 2(b–d), respectively. In the control cases, the individual layer is represented as the homogenized dielectric layer constituting the effective refractive index (method to calculate the effective refractive index can be found in Supplementary Information). A bare PTB7:PC_71_BM layer is used as the reference case and the enhancement factor is defined as the calculated light absorption from individual constituting layer normalized by the reference case. Wavelength dependent response can be clearly resolved in Fig. 2, which is due to the distinctly different periodicity and hole size of each layer. As shown in Fig. 2(b), no obvious absorption peak is found in the cribellum layer. In addition, almost identical absorption spectra can be obtained from the corresponding control case using effective index. As the periodicity of the cribellum layer is significantly smaller than the wavelength of sunlight, the scattered field is predominantly evanescent within the target visible spectrum. Thus, the simulation results suggest that the deep sub-wavelength features of cribellum layer do not contribute significantly to the light trapping effect at visible frequencies and its optical property can be represented using the effective media. The limited enhancement is due to the reduced reflection at the surface on the effective media with the lowered effective refractive index. In contrast, a pronounced enhancement peak centered at 390 nm is found in the cribrum layer (Fig. 2(c)), and a wider peak centered at 750 nm is observed in the internal plate in Fig. 2(d) by comparing to the corresponding control cases. These two peaks can be attributed to scattering by the periodic pattern rather than the contribution of the anti-reflection effect from individual layers^42^,^43^. Therefore, as shown in Fig. 3(a), the diatom frustule is modeled as a stacked system containing cribellum, cribrum and the internal plate, in which the cribellum layer is represented by the homogenized dielectric layer with effective refractive index to save computing resource.

The light trapping effect of the nanostructured diatom frustule being placed on the surface of PTB7:PC_71_BM layer while the bare PTB7:PC_71_BM layer was used as the reference case. The case 1 shown in Fig. 3(a) refers to the configuration in which the internal plate is in direct contact with the active layer. To account for the anti-reflection effects, control case 1 was implemented by representing individual constituting layer using the homogenized effective refractive index. Placing the diatom frustule layer on the top of active layer enhances the absorption efficiency by the factor of 1.41 over the visible spectral range from 380 nm to 800 nm, while the averaged enhancement for the control case 1 is 1.07. The simulation results clearly validate the contribution to the light-trapping effect due to the multiscale structure within the diatom frustule with overall 32% enhancement. As shown in in Fig. 3(a), two prominent enhancement peaks can be identified in the spectrum region from 380 nm to 500 nm and 650 nm to 800 nm with the maximum enhancement factors of 1.73 and 2.18, respectively. The locations of these two peaks (λ_1_ = 400 nm and λ_2_ = 750 nm) agree reasonably well with the absorption peaks in those single layer cases, i.e. 390 nm for cribrum case in Fig. 2(c) and 750 nm for internal plate case in Fig. 2(d). Thus the total enhancement effect of the diatom frustule can be approximated as a combination of contributions from each individual layer. The alternative configuration is also study by reversing the stacking sequence of cribellum, cribrum and the internal plate in both case 2 and control case 2. The resulting absorption spectra are shown in Fig. 3(b). Similarly, broadband enhancement can be found in the spectrum regions from 380 nm to 500 nm and 650 nm to 800 nm, with averaged enhancement factor of 1.38 over the visible spectrum in case 2. In contrast, the corresponding enhancement factor for control case 2 is only 1.10. Two prominent enhancement peaks can be found at the center wavelength of λ_3_ = 430 nm and λ_4_ = 780 nm, which again agree reasonably well with the absorption peaks found in individual layer. The similar light-trapping characteristics of these two cases suggest that the collective light-trapping effect can be treated as the combination of each individual layer’s contribution and is performance is less sensitive to the orientation of the diatom frustule.

To further understand the multiple scattering at the observed enhancement peaks, finite difference time domain (FDTD) simulations (FDTD Solution, Lumerical Inc.) of the diatom frustule were performed. Three-dimensional schematic views of the models showing the cross-section along YZ plane are depicted in Fig. 3. The FDTD simulated absorption spectra in Fig. 4(a,e) reveal that enhanced absorption can be found in the spectral range from 380 nm to 550 nm, 650 nm to 800 nm for case 1, and from 430 nm to 530 nm, 650 nm to 800 nm for case 2, which agrees reasonably well with the RCWA simulated results. The normalized electric-field-intensity |**E/E**_**0**_|^2^ profiles at the corresponding enhancement peaks, λ_b_ = 405 nm and λ_c_ = 700 nm (case 1), λ_f_ = 450 nm and λ_g_ = 720 nm (case 2), are plotted in Fig. 4(b,c,f and g). In all of four plots, the constructive interference resulted from multiple scattering forms the localized “hot-spot” regions that are found to spatially overlap with the 50 nm thick active layer, hence enhancing the absorption within. In comparison, the reduced field overlap with the active region is found at the wavelength (640 nm) corresponding to the dip in the enhancement curve Fig. 4(d,h). Moreover, the FDTD absorption spectra and the normalized electric-field-intensity |**E/E**_**0**_|^2^ profile for the control cases using the featureless dielectric layer constituting effective index are plotted in Supplementary Fig. S4. The marginal improvement of the light absorption is due to the Fabry-perot interference in the thin dielectric layer. The maximum electric field for the control case is less than that of the diatom frustule cases as shown by the color bar and no “hot-spots” forms due to the absence of the scattering. This observation confirms that the diatom frustule pattern can enhance the collection efficiency of the absorption layer, which is consistent with the previous study where dielectric photonic crystal structures were used as the top layer to enhance light trapping in thin-film solar cells^44^.

To explore the influence of the incident angle on the absorption efficiency, which was crucial for device performance at different times of the day, simulations at the progressive increase of the incident angle from 10° to 30° were performed, as shown in Supplementary Fig. S5. The angular dependence of light trapping efficiency for the diatom frustule can be found and the absorption enhancement of the model with diatom frustule (case 1 and case 2) is kept well above that of the control cases under light with varying incident angle, indicating substantial robustness in absorption enhancement of the diatom frustule for a wide range of angles.

Experimental studies were further performed to validate the numerical investigation of the light trapping effects. The sample consists of a 50 nm PTB7:PC_71_BM active layer with cleaned diatom frustules on the top, as sketched in Fig. 5(a). *Coscinodiscus sp*. were obtained from National Center for Marine Algae and Microbiota (NCMA), (Gulf of Maine, North America), and the cultures were maintained at 0–6 °C, using a 13 hour light and 11 hour dark cycle. Guillard’s (F/2) Marine Water Enrichment Solution (Sigma-Aldrich) was used to culture *Coscinodiscus sp*.^45^. Prior to the measurement, a 50 nm PTB7:PC_71_BM layer was spin coated on the glass substrate and maintained in nitrogen environment. Simultaneously, residual organic materials and metal oxides were removed by the cleaning processes that include heating, acid cleaning, and centrifuging^46^. Floating assembling method^46^ was utilized to construct an array of cleaned diatom frustules on the top. In detail, deionized water (50 μL) was added to the planner active layer to form a droplet and then clean frustules of diatom *Coscinodiscus sp*. were dispersed to the water droplet. The diatom frustules were placed on the surface of the active layer upon the evaporation of the water. Finally, both transmission and reflection spectrum of the fabricated samples were measured using Leica DMI 3000M microscope (20 × objective, numerical aperture NA = 0.4, 100W halogen light source) and a matching grating spectrometer (SR-303i, Andor Technology).

The experimentally measured reflection *R(λ)* and transmission *T(λ)* spectra were used to determine the resulting absorption *A(λ)* spectra using the relation *A(λ)* = 1 *–* *R(λ)* – *T(λ)*. The reference sample consists of a bare PTB7:PC_71_BM layer on the glass substrate. The absorption spectra and the enhancement over the reference are shown in Fig. 5(b). Compared with the simulation results, the experimental absorption enhancement curve is relatively flat and no significant modulations exist in the 500–650 nm region. This relatively flat curve represents an average result of diatom frustules with various thicknesses, hole sizes and lattice constants, which are assumed fixed in the simulations, leading to stronger modulations in the optical response. Apart from this, the absorption spectrum for the sample coated with diatoms exhibits broadband enhancement over the reference, and the enhancement factor is 28% above that of the control cases of multi layers with homogenized effective refractive index on top of the active layer. In addition, the peaks of the enhancement lie in the spectrum region from 380 nm to 500 nm and 650 nm to 800 nm, which match closely to the simulation results shown in Fig. 3(a,b). The low absorption of the planar structure beyond 750 nm resulted in an extremely high value of the enhancement factor, similar to the simulation results in Fig. 3. The agreement between experimental and numerical results validates the numerical analysis of the light trapping effect and highlights the applicability of the diatom frustule structure for enhancing the absorption of realistic solar cell devices. Furthermore, more precise calculation can potentially performed by accounting for the statistical variation of the frustule geometry.

In conclusion, the silica frustules of the natural diatom have been modeled as a hierarchical structure consisting micro-/nano-scale features. The optical properties of the diatom frustules acting as the light trapping layer on top of the low bandgap polymer PTB7:PC_71_BM layer were studied numerically using both RCWA and FDTD methods, and broadband enhancement of absorption in the spectrum region from 380 nm to 500 nm and 650 nm to 800 nm was observed. Additional simulations of the case with the reverse stacking sequence suggest that the observed enhancement is mainly due to the multiple scattering induced by each layer, and the dimension parameters of each layer dominate the scattering effect rather than the arrangement sequence of neighboring layers. Moreover, for practical devices, the angular depended response of light trapping efficiency for the diatom frustule was explored and the absorption enhancement was kept well above that of the control cases under light with varying incident angle. The numerical simulations were further validated experimentally by measuring the absorption spectra of active layer with actual diatom frustule on top. Moreover, several self-assembly methods to fabricate monolayer films of irregular micro scale plates with non-uniform sizes have been reported^46^,^47^. It may enable scalable assembly of diatom frustules to form large-scale light-trapping structure at extremely low cost.

## Methods

### Numerical Simulation Methods

**Figure 3:**
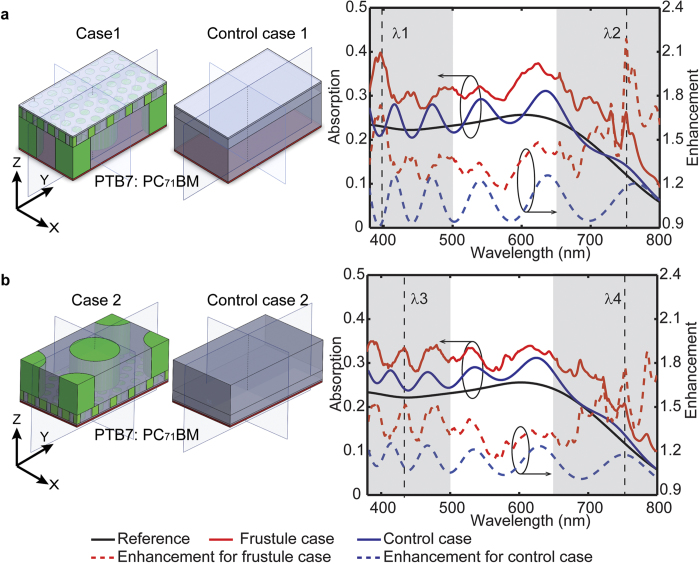
Simulated absorption spectra of the simplified diatom frustule model with the 50 nm thick active layer in direct contact with (**a**) the internal plate and (**b**) cribellum layer, corresponding to case 1 and case 2. The cases of multi layers with homogenized effective refractive index on top of the active layer are used as control cases, corresponding to case 1 and case 2. The case of bare active layer is used as the reference. The enhancement is defined as the ratio of the absorption efficiency between the model with frustule cases (or control cases) and the reference.

Calculations were performed with rigorous coupled wave analysis (RCWA) and finite-difference time-domain method (FDTD). The scattering effect and absorption were calculated by using RCWA method, one of the most commonly used techniques to solve the scattering problem of periodic dielectric structures in Fourier space. For the multilayered dielectric stacks, Fourier expansions of both the field and the permittivity lead to an algebraic eigenvalue system for each layer. The local field distribution was calculated by the FDTD method. In our simulation, FDTD were carried out using Lumerical FDTD Solutions software (http://www.Lumerical.com/). All simulations were for a hierarchical hexagonal array of three dimensional structures with periodic boundary conditions in the x and y directions, as shown in Fig. 3. A broadband (380–800 nm) plane wave source polarized along the x-axis or y-axis was incident from within the glass region.

### Diatom frustule preparation

The cultivated *Coscinodiscus sp*. diatoms were obtained from National Center for Marine Algae and Microbiota (NCMA) (https://ncma.bigelow.org/). Prior to the experiments, residual organic materials and metal oxides were removed by heating the diatoms, which were mixed with adequate amounts of sulfuric acid (H_2_SO_4_) at 80 °C for 20 min. The mixture was centrifuged with 8000 rpm/30 s. Then added enough hydrogen peroxide (H_2_O_2_) into the centrifugal products and heated the mixture at 80 °C for 30 min to remove the residual organic material further. Finally the residual mixture was fully washed with deionized water until pH = 7 and then centrifuged and dried.

### Spectra measurement

For the optical characterization, the transmission spectra from λ = 380 nm to λ = 800 nm are measured using an Andor SR-303i_A spectrometer combined with Leica DMI 3000 M microscope (20 × objective, numerical aperture NA = 0.4, 100 W halogen light source and visible to near-infrared polarizer). Normalization is with respect to the bare glass substrate (for transmittance) and a standard reflector (for reflectance), respectively. The spectrometer measured the reflection *R(λ)*, transmission *T(λ)* and the absorption *A(λ)* was calculated by *A(λ)* = 1 *–* *R(λ)* – *T(λ)*.

## Additional Information

**How to cite this article**: Chen, X. *et al*. Numerical and experimental investigation of light trapping effect of nanostructured diatom frustules. *Sci. Rep*. **5**, 11977; doi: 10.1038/srep11977 (2015).

## Supplementary Material

Supplementary Information

## Figures and Tables

**Figure 1:**
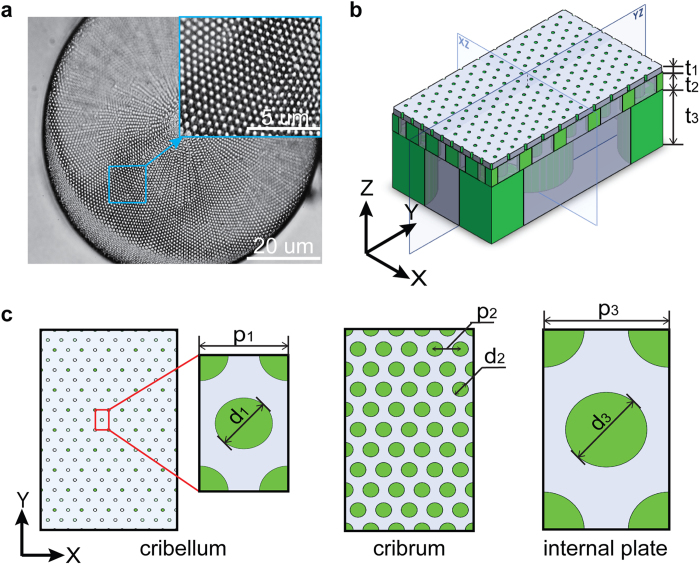
(**a**) Optical microscopy images of *Coscinodiscus sp*. (**b**) Simplified 3D structure of the unit cell of diatom frustule based on experimental results. Thickness of the three layers: Cribellum t_1_ = 50 nm, Cribrum t_2_ = 300 nm, Internal Plate t_3_ = 1000 nm, (**c**) Left: top view of cribellum, the lattice constant p_1_ = 200 nm and the hole size d_1_ = 50 nm. Middle: top view of cribrum, the lattice constant p_2_ = 400 nm and the hole size d_2_ = 250 nm. Right: top view of internal plate, the lattice constant p_3_ = 2 μm and the hole size d_3_ = 1.3 μm.

**Figure 2:**
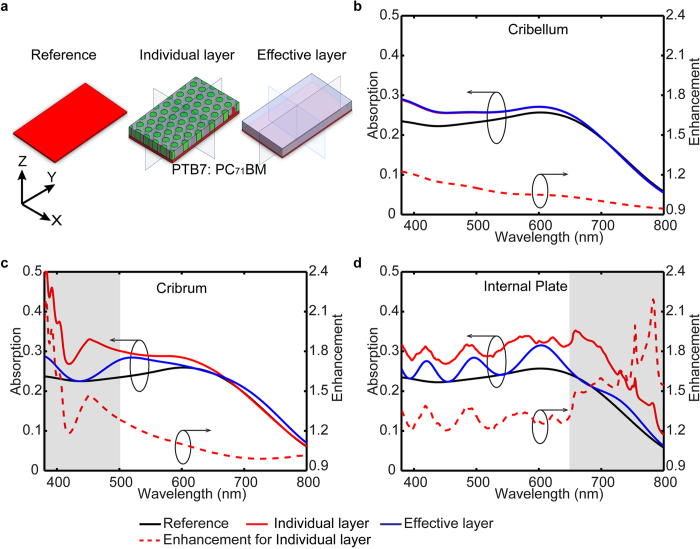
RCWA simulated absorption spectra of the individual constituting layer (cribellum, cribrum and the internal plate) being placed on the surface of a 50 nm thick active layer (PTB7: PC_71_BM). The case of the effective layer (homogenized dielectric layer with effective refractive index corresponding to each layer) being placed on active layer was used as control case, and the case with bare active layer is used as the reference. The enhancement is defined as the ratio of the absorption efficiency between the model with individual constituting layer and the reference.

**Figure 4:**
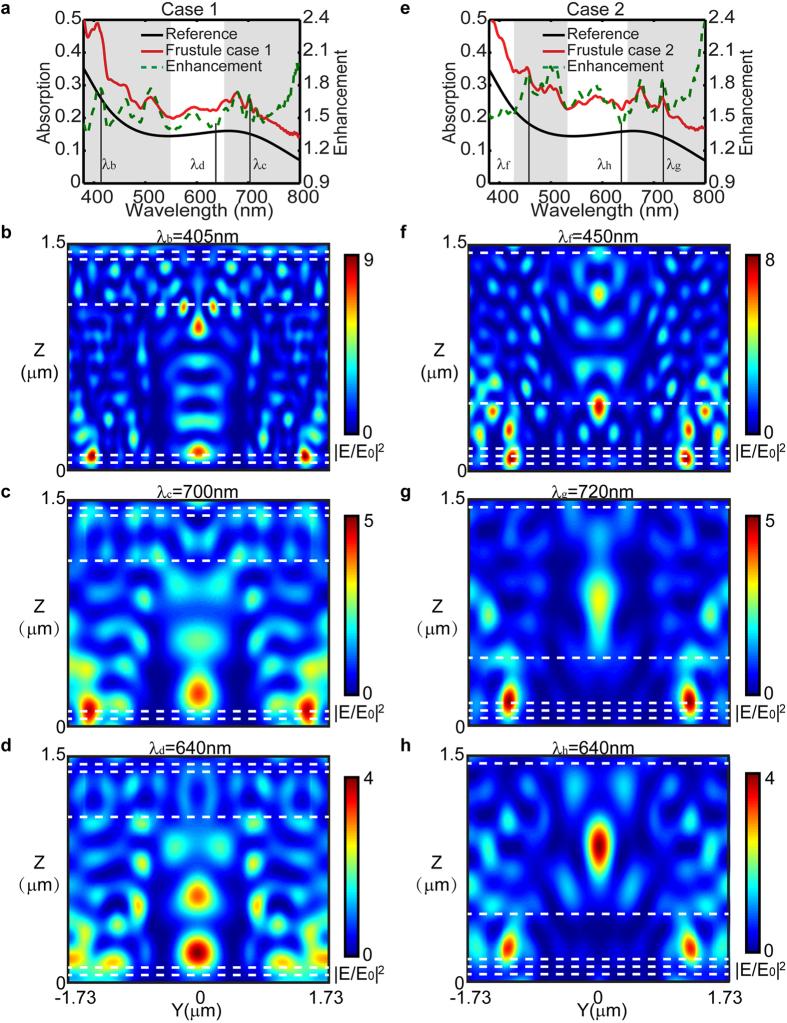
FDTD simulated absorption spectra of the simplified diatom frustule model with the 50 nm thick active layer, case 1 (**a**) and case 2 (**e**). And normalized electric-field-intensity |**E/E**_**0**_|^2^ distribution on the Y-Z plane at λ_b_ (**b**), λ_c_ (**c**), and λ_d_ (**d**) for case 1, λ_f_ (**f**), λ_g_ (**g**), and λ_h_ (**h**) for case 2. Stacking sequence of case 1 (From top to bottom: air, cribellum, cribrum, internal plate, active layer and substrate), stacking sequence of case 2 (From top to bottom: air, internal plate, cribrum, cribellum, active layer and substrate).

**Figure 5:**
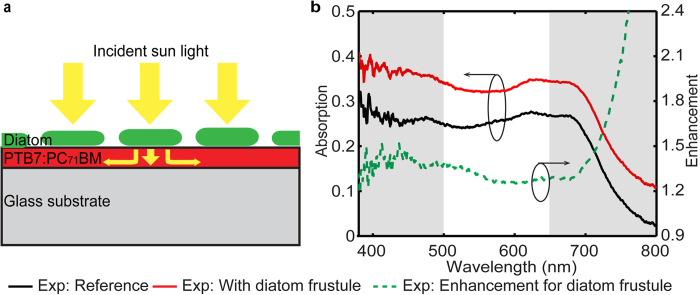
(**a**) Schematic representation of a PTB7:PC_71_BM based thin film with the diatom frustules on top layer. (**b**) Experimental absorption spectrum for the model of planar active layer without diatom frustule on top (black curve) and with diatom frustule (red curve), with the experimental absorption enhancement curve for diatom frustule (green dashed curve).
