# Supplementary material for: Numerical and experimental investigation of light trapping effect of nanostructured diatom frustules
Source: Sci Rep. 2015 Jul 9;5:11977. doi: 10.1038/srep11977 (PMC4496668; doi:10.1038/srep11977)
Supplement: Supplementary Information [file srep11977-s1.pdf]

Supplementary Information

Numerical and experimental investigation of light trapping effect of nanostructured diatom frustules

Xiangfan Chen, Chen Wang, Evan Baker and Cheng Sun†

Mechanical Engineering Department, Northwestern University, Evanston, IL 60208, USA

† To whom correspondence should be addressed. E-mail: c-sun@northwestern.edu

1. Material properties used in the numerical simulation

The dispersive refractive index of SiO_2 and PTB7: $PC_{71}BM$ were obtained from the experimental data[1,2], as shown in Fig. S1(a) and Fig. S1(b), respectively. The holes in the diatom frustules were filled with air with the refractive index $n_{air}=1$.

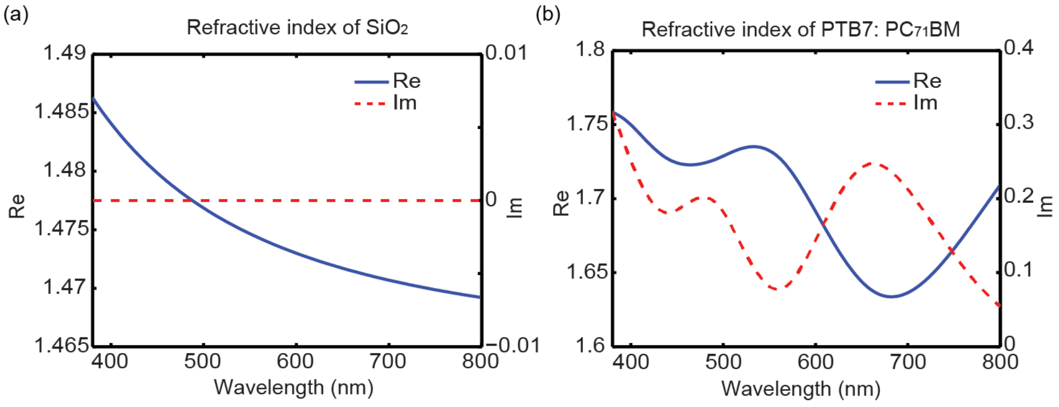

Figure S1. Dispersive refractive index of (a) SiO_2 and (b) PTB7: $PC_{71}BM$.

2. Effective refractive index

For the simplification in simulation, individual constituting layer of diatom frustule is simplified as a homogenized dielectric layer under effective media approximation (defined as effective layer) with effective refractive index n_{eff} (Eq. (1))[3].

$$n_{eff} = f \times n_{air} + (1 - f) \times n_{SiO_2} \quad (1)$$

f is the volumetric filling ratio of the holes.

3. Polarization-dependent absorption characteristics for individual constituting layer

For convenience, incidence with electric field along the x-axis and y-axis are referred as X polarized and Y polarized incidence, respectively, as shown in Fig. S2(a), (b).

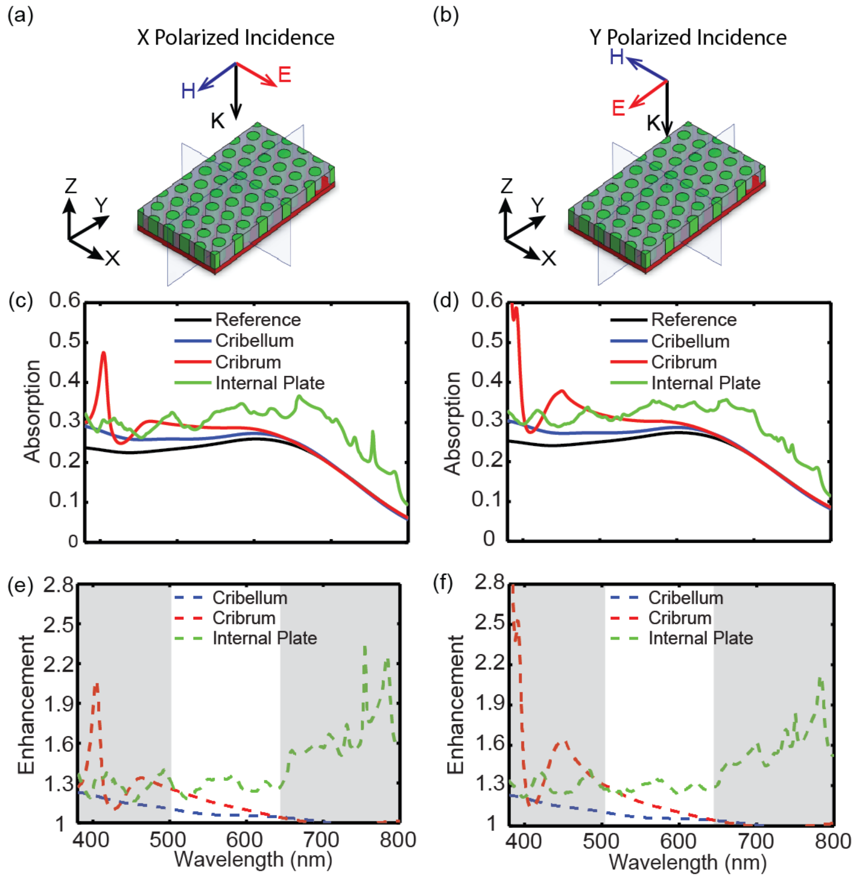

Figure S2. Simulated absorption spectra of individual constituting layer (cribellum, cribrum, and internal plate) of the diatom frustule being placed on the surface of a 50 nm thick active layer (PTB7: $PC_{71}BM$) under (a) X polarized incidence and (b) Y polarized incidence. The case with bare active layer is used as the reference. The enhancement is defined as the ratio of the absorption efficiency between the model with individual constituting layer and the reference.

4. Polarization-dependent absorption characteristics of diatom frustule

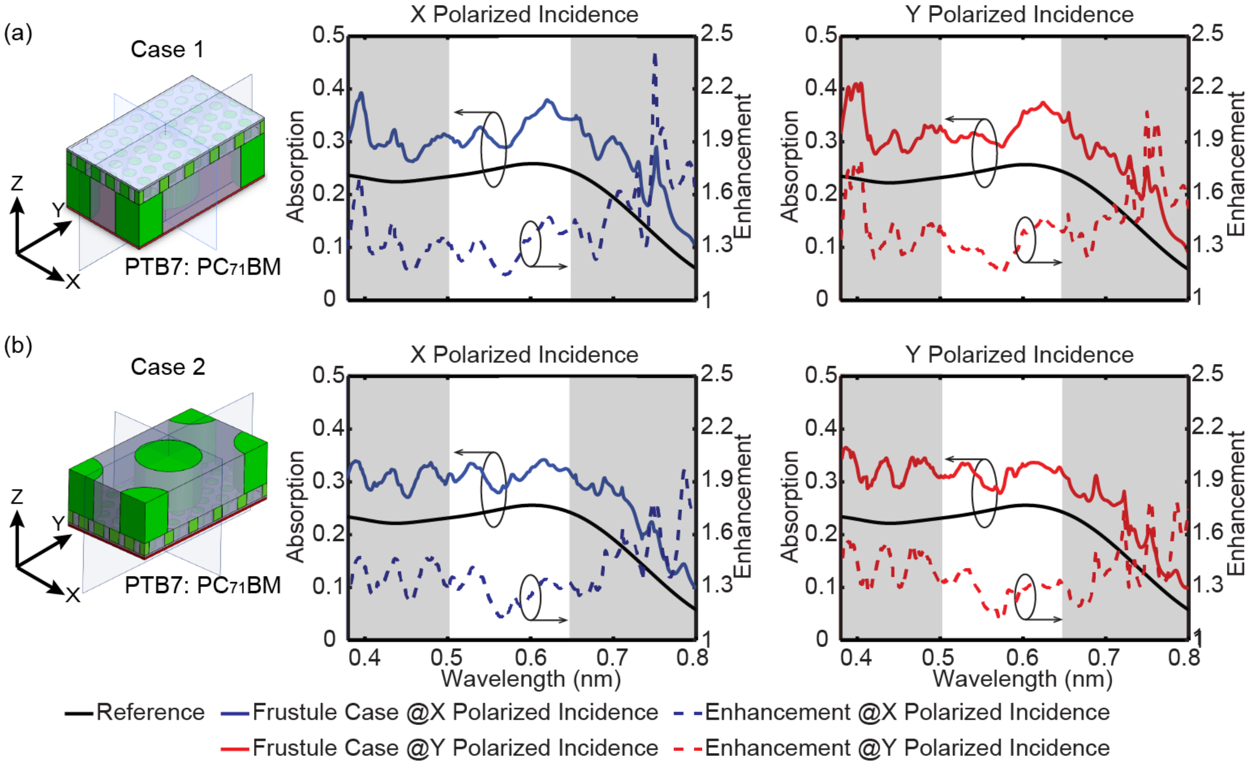

Figure S3. Simulated absorption spectra (X and Y polarized incidence) of the simplified diatom frustule model with the 50 nm thick active layer in contact with (a) the internal plate and (b) cribellum layer. The case with bare active layer is used as the reference. The enhancement is defined as the ratio of the absorption efficiency between the model with frustule cases and the reference.

5. Electric field distribution for control cases

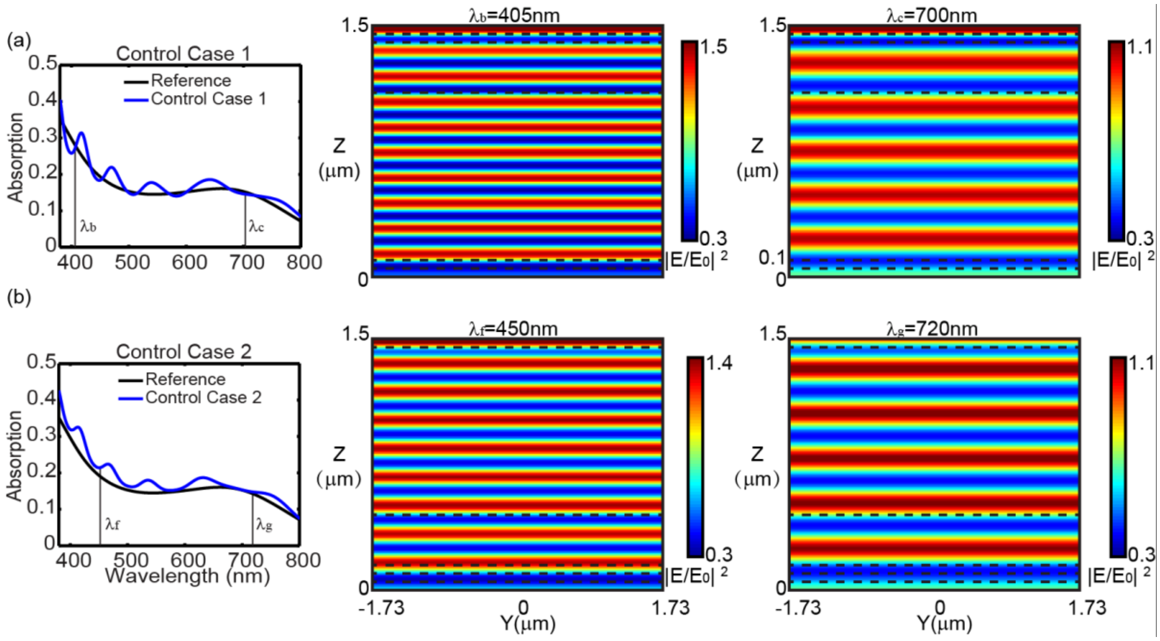

Figure S4. FDTD simulated absorption spectra of the multi effective layers with the 50 nm thick active layer in contact with (a) the effective internal plate (control case 1), and (b) effective cribellum layer (control case 2). And normalized electric-field-intensity $|\mathbf{E}/E_0|^2$ distribution on the Y-Z plane at λ_b=405 nm, λ_c =700 nm for control case 1, and λ_f=450 nm, λ_g=720 nm for control case 2. Stacking sequence of control case 1 (From top to bottom: air, cribellum, cribrum, internal plate, active layer and substrate), stacking sequence of control case 2 (From top to bottom: air, internal plate, cribrum, cribellum, active layer and substrate).

6. RCWA simulation for angular response

To explore the influence of the incident angle on the absorption efficiency, which was crucial for device performance at different times of the day, simulations at the progressive increase of the incident angle from 10° to 30° were performed, as shown in Fig. S5. The angular dependence of light trapping efficiency for the diatom frustule can be found and the absorption enhancement of

the model with diatom frustule (case 1 and case 2) is kept well above that of the control cases under light with varying incident angle. For example, when the incident angle θ=30°, case 1 shows absorption enhancement by the factor of 1.52 over the visible spectral range, while the average enhancement factor for the control case 1 is 1.02. Moreover, for case 1, substantial enhancement can be identified in the spectrum region from 450 nm to 600 nm and 650 nm to 800 nm with the maximum enhancement factors of 1.69 (at 500nm) and 2.17 (at 770nm), respectively.

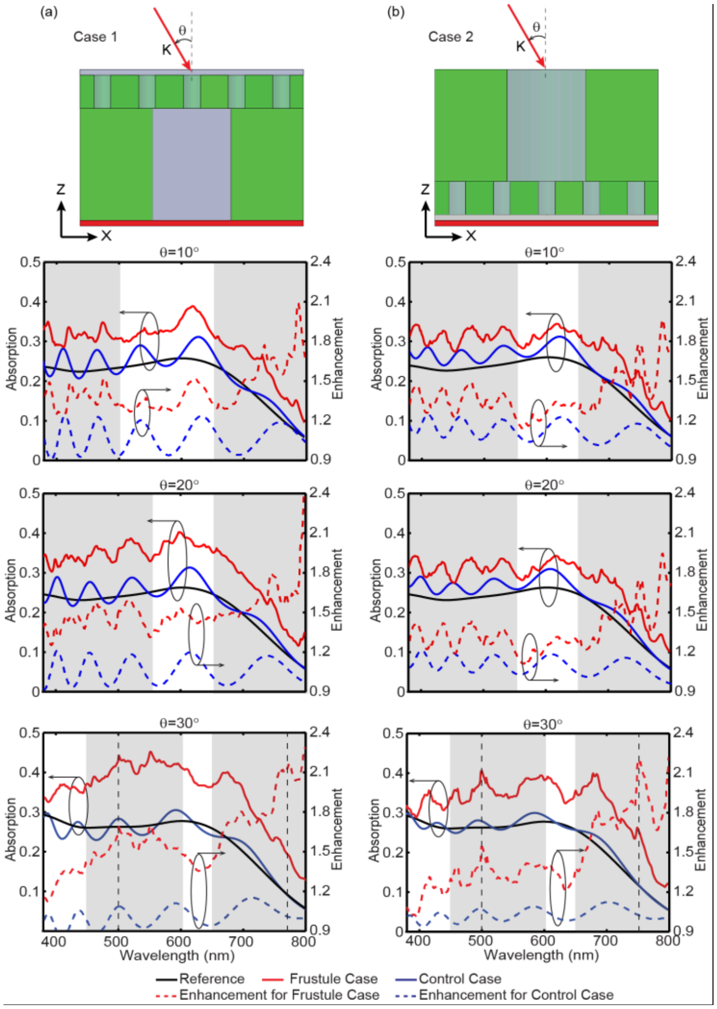

Figure S5. RCWA simulated absorption spectra (average of X and Y polarized incidence) of case 1 (a) and case 2 (b) under oblique incidence (θ=10°, 20°, 30°).

7. Comparison of experimental results with simulation results

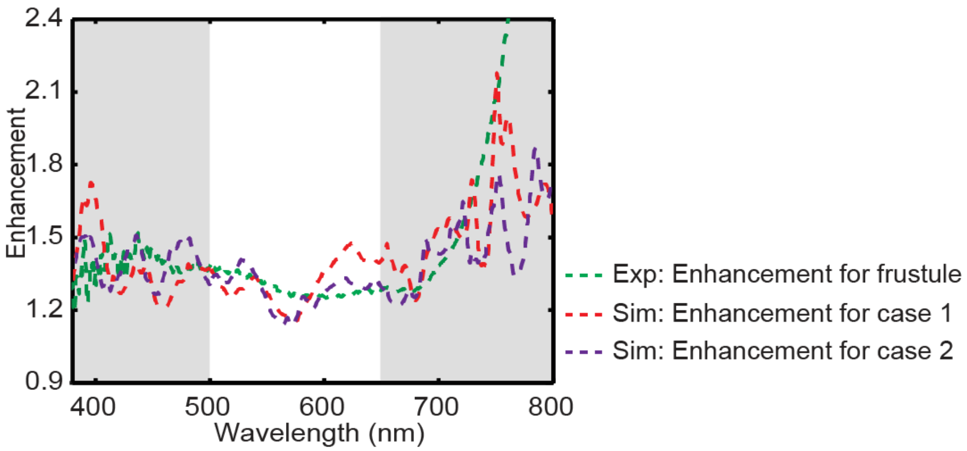

Figure S6. Comparison of absorption enhancement factor of diatom frustule between experimental results with simulation results. The experimental absorption enhancement curve for diatom frustule (green dashed curve), and simulated absorption enhancement for case 1 (red dashed curve) and case 2 (purple dashed curve).

Reference:

1. Gao, L. H., Lemarchand, F. & Lequime, M. Exploitation of multiple incidences spectrometric measurements for thin film reverse engineering. *Opt Express* **20**, 15734-15751,(2012).
2. Liang, Y. Y. & Yu, L. P. A New Class of Semiconducting Polymers for Bulk Heterojunction Solar Cells with Exceptionally High Performance. *Accounts Chem Res* **43**, 1227-1236,(2010).
3. Braun, M. M. & Pilon, L. Effective optical properties of non-absorbing nanoporous thin films. *Thin Solid Films* **496**, 505-514,(2006).
